# Characterizing innovators: Ecological and individual predictors of problem-solving performance

**DOI:** 10.1371/journal.pone.0217464

**Published:** 2019-06-12

**Authors:** Sanjay Prasher, Julian C. Evans, Megan J. Thompson, Julie Morand-Ferron

**Affiliations:** Department of Biology, University of Ottawa, Ottawa, Ontario, Canada; Office National de la Chasse et de la Faune Sauvage, FRANCE

## Abstract

Behavioural innovation, the use of new behaviours or existing ones in novel contexts, can have important ecological and evolutionary consequences for animals. An understanding of these consequences would be incomplete without considering the traits that predispose certain individuals to exhibit innovative behaviour. Several individual and ecological variables are hypothesized to affect innovativeness, but empirical studies show mixed results. We examined the effects of dominance rank, exploratory personality, and urbanisation on the innovativeness of wild-caught black-capped chickadees using a survival analysis of their performance in two problem-solving tasks. Additionally, we provide one of the first investigations of the predictors of persistence in a problem-solving context. For lever pulling, we found a trend for dominants to outperform subordinates, particularly in rural birds, which did not align with predictions from the necessity drives innovation hypothesis. When examining possible explanations for this trend we found that older chickadees outperformed younger birds. This follow-up analysis also revealed a positive effect of exploratory personality on the lever-pulling performance of chickadees. Our results suggest that experience may foster innovation in certain circumstances, for instance via the application of previously-acquired information or skills to a novel problem. As we found different predictors for both tasks, this suggests that task characteristics influence the innovative propensity of individuals, and that their effects should be investigated experimentally.

## Introduction

Animal innovation, defined as the use of novel behaviours to meet challenges or the application of existing behaviours to solve novel problems [1], has been shown to play an important role in the ecology and evolution of diverse taxa. For instance, avian species characterized as successful invaders of novel environments tend to have higher innovation rates than unsuccessful species [2]. Additionally, avian taxa with high innovation rates were found to contain a greater number of species [3], and at the intra-specific level, innovative behaviour has been associated with increased reproductive success [4–7]. Despite the research effort prompted by these implications, the literature on the predictors of individual innovativeness shows mixed results [8], and the question of which characteristics make some individuals more innovative than others remains.

The “necessity drives innovation” hypothesis suggests that individuals will be more likely to exhibit innovative behaviour when resources are scarce [9,10]. Animals with a greater necessity for resources are expected to more readily approach and interact with tasks compared to less motivated individuals [8]. Subordinates may have a greater necessity to innovate than dominant individuals and they may do so as an alternative to competing with dominant individuals for familiar resources. This pattern has been found in chimpanzees (*Pan troglodytes*, [11]), meerkats (*Suricata suricata*, [12]), and great tits (*Parus major*, [13]). However, dominance rank was found not to influence problem-solving performance in pigeons (*Columba livia*, [14]) or spotted hyenas (*Crocuta crocuta*, [15]). An individual’s necessity to innovate may also be reflected in the extent of its interaction with a novel problem, such as the number of attempts made or time spent manipulating a task (i.e. its persistence, defined as task-directed motivation [8]). Greater persistence in solving a problem would be expected to increase the amount of information gathered about the problem [16] as well as increase the chance of solving the task by trial and error.

Novelty responses may impact an individual’s innovative potential by affecting its likelihood of being exposed to novel items or locations, and influencing the chances of gaining enough information to reach a solution [8,10]. These responses include object neophilia (the propensity to approach and interact with novel objects), spatial exploration (the speed and extent of an individual’s movement through a novel space), and object neophobia (the tendency to avoid novel objects) [8]. Conflicting results have been reported concerning the influence of novelty responses on individual innovativeness. For instance, more spatially exploratory and less neophobic carib grackles (*Quiscalus lugubris*) were found to outperform less exploratory and more neophobic individuals in an innovation task [17]. Negative relationships between neophobia and innovativeness have also been shown in pigeons and common mynas (*Acridotheres tristis*;[14,18]). On the other hand, some studies have found no relationship between novelty responses and problem-solving performance, suggesting that these traits can vary independently in some species [19–21].

Habitat type, in particular the extent of urbanisation of the environment, is an external factor that has been explored in the context of intra-specific variation in problem-solving performance. Urban environments may contain fewer predators and more ecologically-novel resources [22]. Thus, compared to rural individuals, animals in urban environments would be expected to exhibit less neophobia and a greater inclination to explore, which in turn is expected to increase their innovative performance. A study on common mynas [23], and one on mountain chickadees (*Poecile gambeli*, [24]), provide support for this idea. However, a recent study on house sparrows found that urban birds were no more likely to solve multiple problem-solving tasks compared to rural birds, perhaps reflecting aspects of urban environments that may reduce the tendency to innovate, such as new predators or toxins, and an abundance of accessible food [25]. This study further demonstrates the complexity of the relationship between urbanisation and problem-solving performance as it also found a significant interaction between urbanisation and body mass. Urban birds were more successful than rural birds on the most difficult task only if they had relatively larger body mass [25]. The complex ways in which urban environments vary prevent the development of clear predictions without more information on the characteristics of the environment from which the study subjects originate, and how the subjects might experience the environment based on the species’ ecology [26]. Our prediction for the effect of urbanisation here is based on the assumption that urban environments promote innovation due to the abundance of evolutionarily novel features.

We investigated the effects of dominance rank, exploratory tendency, and urbanisation on the individual problem-solving performance of wild-caught black-capped chickadees (*Poecile atricapillus*) using two distinct foraging tasks. Problem-solving performance serves as a proxy for innovation, with birds required to complete each step of the innovation process to successfully solve a task (i.e. discovering the problem, contacting the task, and interacting with it to reach a solution). The black-capped chickadee belongs to Paridae, an avian family showing a high number of innovations in the wild [27], and is a widespread North American species occurring in rural and urban habitats [28,29]. In the non-breeding season, members of this species form stable groups with linear dominance hierarchies [29,30]. We predicted that subordinates would be more successful and faster problem solvers than dominant birds. Additionally, we expect individuals that are faster to explore a novel environment, and/or those that originate from more urbanised habitats, to be more successful and faster when solving tasks compared with slower explorers or birds from less urbanised habitats. Moreover, we provide an examination of individual and ecological determinants of persistence during problem-solving assays. The predictors of persistence are very poorly understood, despite its role in problem solving in various species (e.g. [12,17,18]), and its key importance in determining expertise, which itself can impact survival and reproductive success [31]. To this end, we examine dominance, exploration, and habitat urbanisation as potential predictors of the frequency of contacts made with the tasks.

## Methods

### Subjects and housing

**Fig 1:**
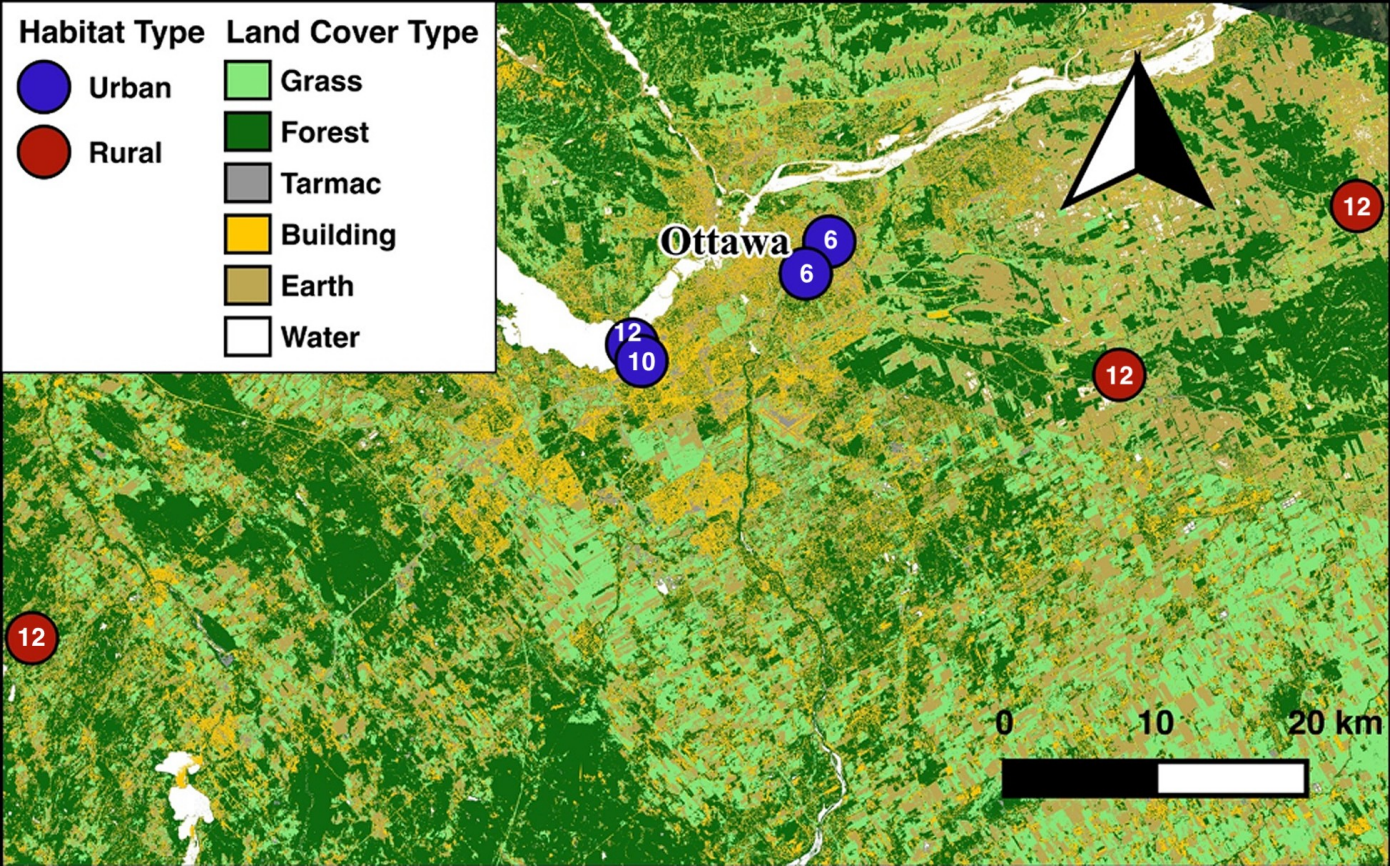
Land cover classification map showing the 4 urban (blue) and 3 rural (red) sites located in and around Ottawa, Ontario, Canada.

From October to December 2016, a maximum of 12 birds were captured weekly using mist nets from one of seven sites in and around Ottawa, Ontario (Fig 1). The degree of urbanisation at each site was assessed using remote sensing data [32–35] by quantifying the number of pixels classified as different land cover types (building, tarmac, forest, and bare earth) within a 1 km radius of capture sites. These variables were used in a principal components analysis (PCA) to generate a unique urban score that explained the degree of urbanisation at each site (81.82% of variance explained by PC1, [36]). Urban score was replaced with a binary habitat variable (urban/rural) in our final models to assess the robustness of our conclusions. Urban sites were urban parks surrounded by houses and located no more than 10 km from downtown Ottawa, and rural sites were forested areas in a rural landscape at least 25 km from downtown. These models returned similar conclusions as those including the continuous urban score (S1 Table).

Upon capture, each individual was fitted with metal and coloured leg bands as well as a unique passive integrated transponder (PIT) tag. Birds were categorized as juvenile (bird born in the previous spring) or adult (more than one year old) by inspecting the shape and wear of their tail feathers [37]. After capture, birds were transported and housed in individual cages, allowing only auditory contact between individuals, in the animal care facility of the University of Ottawa. Outside of testing periods, birds were given ad libitum access to food (sunflower seeds) and water, and mealworms at the end of each day. On the last day in captivity, before being released back at their site of origin, blood samples were taken from the brachial vein of each subject for molecular sexing [36,38]. Subjects were released at their site of capture after a total of 5 days at the university.

### Problem-solving trials

**Fig 2:**
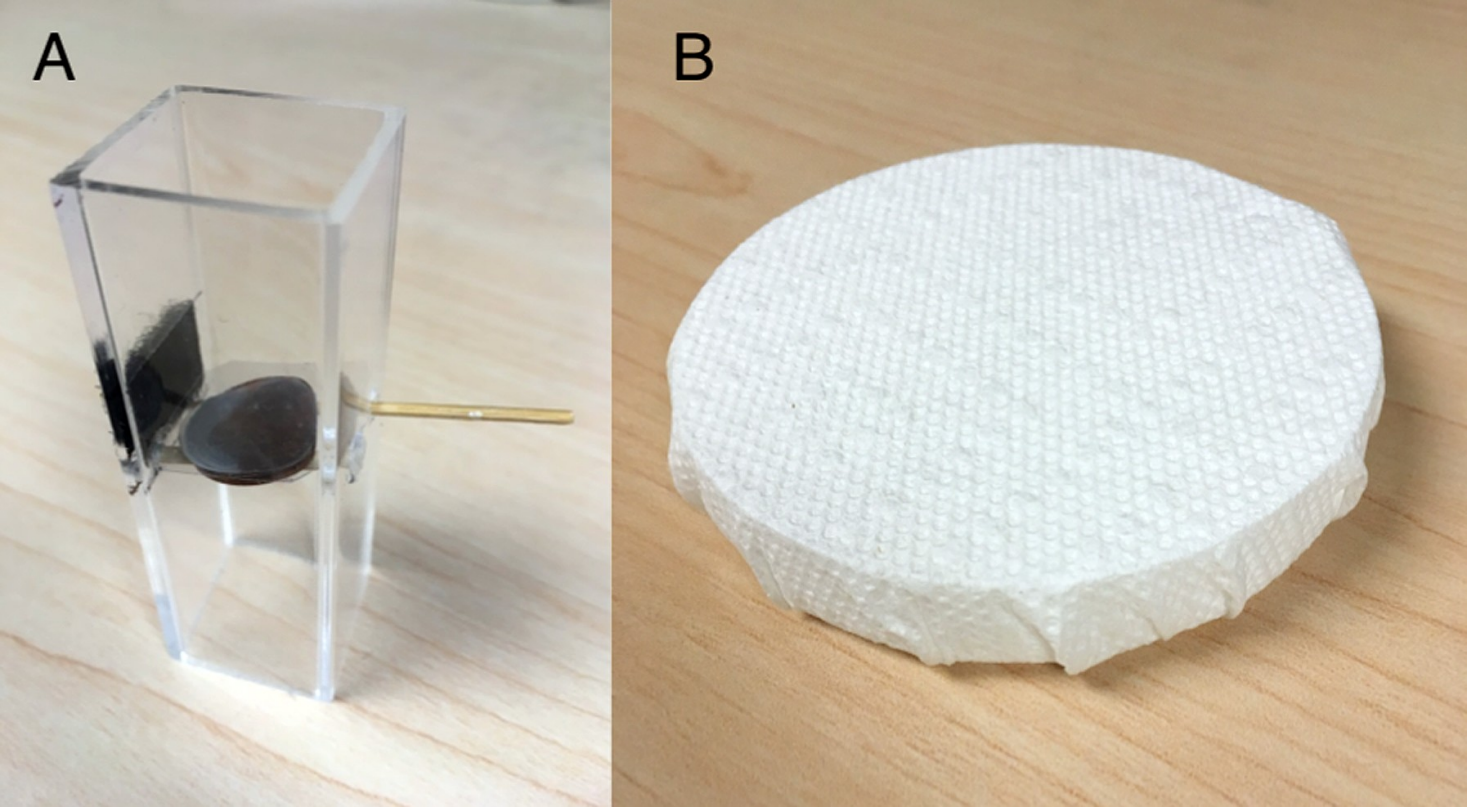
Two extractive foraging tasks used to measure problem-solving performance and persistence in chickadees. (A) The lever-pulling task required birds to pull a lever to cause a platform holding a food reward to fall out of the tube. (B) The paper-ripping task required birds to rip through the paper towel wrapping a Petri dish to gain access to the reward inside.

In captivity, each bird underwent two problem-solving trials for each of two extractive foraging tasks that required the use of different motor actions to solve. The lever-pulling task consisted of a small Perspex tube in which two wax worms were held on top of a platform supported by a lever (Fig 2A; similar to [19]). To reduce accidental solutions the lever was placed in the task at a slight downward angle. Birds were required to pull the lever completely out of the tube, causing the food reward to fall out. The paper-ripping task consisted of the bottom half of a Petri dish, containing seeds and mealworms, wrapped with white paper towel (Fig 2B; similar to [39]). This task was solved when a bird ripped a hole through the paper that was big enough to extract a seed or worm. Birds were expected to be motivated to search for food in the opaque paper-ripping task, as the mealworms they received at the end of each day in captivity were presented in Petri dishes placed in the same location as the task. However, food deprivation periods were used prior to paper-ripping trials to increase the likelihood of birds interacting with this task (see below).

Lever-pulling trials took place on day 1 in captivity from 15:15 to 16:15 and day 2 from 15:00 to 16:00. Birds were not food deprived beforehand and a seed dish remained available during these trials. However, no worms other than those placed in the task were available. Paper-ripping trials occurred on day 3 from 14:15 to 15:15 and day 5 from 7:00 to 8:00. Birds were food deprived for 30 minutes prior to the first paper-ripping trial, and overnight (14 hours) prior to the second. At the start of each trial an experimenter turned off the lights of the housing room, entered with a red headlamp, and fixed a single task in each bird’s cage. The trial began after the experimenter exited the room and turned the light back on. Differences in duration of food deprivation periods resulted from the accommodation of concurrent studies [36,40]. All trials were video recorded for subsequent coding of behavioural data.

Each bird’s performance and persistence in the problem-solving trials were quantified using data extracted from video recordings. Performance was quantified as the success or failure in solving the task and the latency to solve the task, measured as the time from the beginning of the trial to the time of solution. Taking the full latency in this way allows us to capture the entire innovatory process, from the time that an animal takes to first detect the task to the time needed to find a solution. For birds that failed to solve the task in their first trial, but succeeded in their second, we added the duration of their first trial (~60 minutes) to their latency to solve in the second, effectively treating the two trials as a single extended test. Birds that did not solve in the time given were assigned latencies equal to the sum of the two trial durations (~120 minutes). We use the term ‘performance’ when discussing our analyses and results as our main method of analysis takes both the latency to solve and the success or failure of each bird into account (see analysis section). Persistence was quantified using the BORIS software [41] as the number of bill and foot contacts made with the task until it was solved or until the end of the observation period (Intra-observer reliability: all Pearson’s r > 0.988, Inter-observer reliability: all Pearson’s r > 0.776). As with performance, the total number of contacts made with the task was added over both trials for birds that solved in the second trial or did not solve at all. Only performance and persistence data leading up to an individual’s first solution were used for analysis, so as to consider only those instances of problem solving that reflect innovative behaviour.

### Dominance rank

Each site was equipped with a sunflower seed feeder two to seven weeks after releasing birds from captivity and they remained until the end of April 2017. The feeders were fitted with a single perch that restricted access to one individual at a time. These perches contained radio-frequency identification (RFID) antennae (Priority 1 Design, Australia), which recorded the arrival and departure of visits by PIT tagged individuals. We extracted displacement events automatically from these data and assigned each individual a dominance rank depending on the number of times they displaced others or were displaced themselves [42]. A displacement was considered to have occurred when a bird left within one second of another individual landing on the feeder and the newly arrived bird stayed for a minimum of five seconds. Only interactions between tagged chickadees occurring on the feeders could be detected. This method of calculating dominance automatically has been shown to correlate well with traditional methods of measuring dominance based on interactions extracted from video [42]. A linear model analysing age and sex as predictors of dominance scores, while controlling for site as a fixed term (as the model returned zero variance for site when included as a random effect), revealed that males have significantly higher dominance scores than females (F_1,26_ = 12.783, P = 0.001) and adults have significantly higher scores than juveniles (F_1,26_ = 8.836, P = 0.006). This is in agreement with previous findings of the correlates of social rank in chickadees [29,43,44]. As some birds within a group did not interact with others on our feeders, we could not determine the overall linearity of relationships within groups of chickadees [45]. When determining the transitivity of triads (i.e. the linearity of relationships between sets of three individuals that all interacted, A>B>C) at each site using the methods of Shizuka & McDonald [45,46] we found that all triads were transitive at three of the seven sites (P_t_ = 1, t_tri_ = 1), there were significantly more transitive triads than expected at an additional two sites (mean P_t_ = 0.929, mean t_tri_ = 0.715, P < 0.05), and the remaining two sites had significantly less transitive triads than expected (mean P_t_ = 0.393, mean t_tri_ = 1.429, P > 0.39). The lack of transitive triads probably reflects the fact that few individuals were detected at these two sites. We reran our statistical models that included dominance after removing birds from these last two sites to verify that our conclusions were not influenced by their inclusion. Conclusions for problem-solving performance and the influence of dominance were unchanged (S3 and S4 Tables). However, after removal of data points from these two sites, urbanisation no longer appeared in the top models for lever-pulling persistence (n = 34) and it no longer seemed to influence paper-ripping persistence (n = 32, S4 Table).

### Exploration in a novel environment

A spatial exploration assay was conducted on the third day in captivity to measure each individual’s exploratory tendency in a novel environment. The novel room, containing four artificial trees, was accessible to birds from their home cages through an opaque sliding door. Light manipulation was used to move birds to and from the novel room, which avoided handling by the experimenter [47]. Each bird’s number of visits to trees, number of visits to other features in the room, and the duration of their flights and hops were measured over a 10-minute period [48]. A composite exploration score for all birds tested in this season, reflecting activity levels and willingness to explore a novel area, was generated by including these measures in a principal components analysis (similar to [48]). Only the first principal component had an eigenvalue greater than one (with variable loadings ranging from 0.54–0.95) and it explained 75.81% of the variance (S11 Table). A similar measure of spatial exploration was found to be moderately repeatable after exposing each chickadee to a second exploration assay (on day 4) with new artificial trees placed in different positions (N = 70, R = 0.47, CI = 0.41–0.51; [36]).

### Analysis

When analysing the predictors of problem-solving performance, we conducted an extended cox proportional hazards regression. This is a semi-parametric survival analysis approach that makes no assumptions about the distribution of the response variable [49]. We used this approach as an alternative to assigning capped latencies to individuals that did not solve a problem, which would suggest that the bird solved at that time and may not allow the data to meet assumptions of normality for commonly used regression analyses. Using censored observations allows us to avoid those assumptions and incorporate our limited knowledge of each bird’s performance on a task (e.g. we only know that an unsuccessful bird was not able to solve a task in the time given). Cox proportional hazards regression has been used to analyse problem-solving performance by multiple researchers (e.g. [23,25,50]). The ‘extended’ version of the analysis includes time-dependent covariates (see below), which are variables whose value for a particular subject changes over the course of the study [51]. We coded individuals that failed to solve during the experiment as censored observations, since we do not know their true latency to solve (e.g. [23]). For instances in which birds solved with wings, or worms escaped from the lever-pulling device (11/70 birds), we also assigned censored latencies up to the time of the incident. Individuals that had retrieved cached seeds during the trials (10/70 birds, all during paper-ripping) were excluded from paper-ripping analyses.

We built a separate extended cox proportional hazards model for each task, setting the latency to solve as the response variable and capture site as a random intercept. We examined the influence of dominance, exploration, and urbanisation scores on the problem-solving performance of birds in each task. An interaction term between dominance and urbanisation was included in each model to account for the possibility that urbanisation may influence the social dynamics of chickadees [52], and modify the impact of dominance on innovative tendencies. As persistence is often positively associated with problem-solving performance (e.g. [12,15,18,25,53–55]), we controlled for the number of contacts made with the task throughout the two trials (persistence) as a time-dependent covariate. Our measure of persistence fits the definition of a time-dependent covariate, because the number of contacts made with a task increased as the trials progressed. When analysing a time-dependent covariate, the study period is divided into time intervals and the time-dependent covariate has a different value in each interval. The cox analysis proceeds by comparing the value of the variable when a bird has solved a task to the value of that variable for other individuals in the same time interval. Since a bird necessarily has to contact a task in the time interval in which it solves, and it is unlikely that other birds are contacting their tasks as frequently in the exact same time interval, this statistical approach is expected to produce an effect of persistence on solving performance. As a result, we will not make conclusions based on the significance of this variable, but will treat it only as a confounding variable by including it in all models.

In the case of the lever-pulling task, urbanisation score needed to be converted to a categorical variable (urban/rural) and stratified to meet the proportional hazards assumption [49]. Stratification of a variable allows it to be controlled for, but prevents the model from returning an estimate. Inspection of the DFBETA residual plots (showing the impact of each observation on the model estimates) for each of the predictors allowed us to identify an influential observation (having a residual greater than 1; [56]), and the lever-pulling analysis was conducted after removing it. The time-dependent contacts variable failed to meet the proportional hazards assumption for the paper-ripping task, so these data were analysed after excluding three influential observations. Results of analyses before removing influential observations are provided in the supplementary information (S2 Table). The random intercept of site was removed from each model as it was non-significant in likelihood ratio tests (lever pulling: χ^2^ = 0.119, df = 1, *P* = 0.731; paper ripping: χ^2^ = 0.001, df = 1, *P* = 0.971).

Following the precautions taken to meet model assumptions, a set of models with all combinations of the predictors was generated using the dredge function of the R package ‘MuMIn’ [57]. Model averaging was conducted on the subset of these models that were within 2 AICc units of the best fitting model [58]. Model averaged estimates, calculated using the ‘zero method’ [59], were used to ascertain the relative effects of our variables on performance in each task.

Following the results gained from the analyses above, we built an additional model to investigate predictors underlying the association between dominance and lever-pulling performance. We replaced the dominance variable with age and sex in this new model and added an interaction between each of these and urbanisation. Removing dominance increased our sample size by 13 individuals (33.33%), because some birds studied in captivity were not subsequently detected in displacement interactions at the feeders in the winter. We then conducted model selection and averaging to understand the impacts of age and sex on lever-pulling performance.

When analysing the characteristics associated with persistence, we used each bird’s total number of contacts (until the task was solved or the observation became censored) as our response variable. Scores for dominance, exploratory tendency, and urbanisation were used as predictors in generalized linear mixed models with a negative binomial error structure to control for overdispersion [60]. Additionally, an interaction term between dominance and urbanisation score was included for both tasks. We controlled for each bird’s latency to solve or become censored as a fixed term, and their site of capture was included as a random intercept. We conducted the same steps for model selection and averaging as before to reach conclusions on the predictors of persistence.

To determine whether individuals performed consistently across tasks, we calculated the correlation-based repeatability of problem-solving latencies and overall persistence. Kendall’s tau-b correlations are reported to account for violations of bivariate normality and for ties in problem-solving efficiency and persistence [61]. We also use a chi-square test of independence to investigate whether success in solving one task is associated with success in the other.

All continuous predictors were standardized (rescaled between 0 and 1, and mean-centred) prior to running survival and generalized linear mixed models [62]. Cox models were built using the ‘coxme’ [63] and ‘survival’ [49,64] packages in R, while the generalized linear mixed models were created using the ‘lme4’ package [65]. All statistical analyses were completed using R version 3.4.3 [66].

### Ethics statement

The University of Ottawa Animal Care Committee (protocols 1758–59) approved this study, which was also completed in accordance with the regulations of and under scientific (SC-42) and banding permits (10854) from Environment Canada, and the Canadian Wildlife Service.

## Results

### Lever pulling

**Fig 3:**
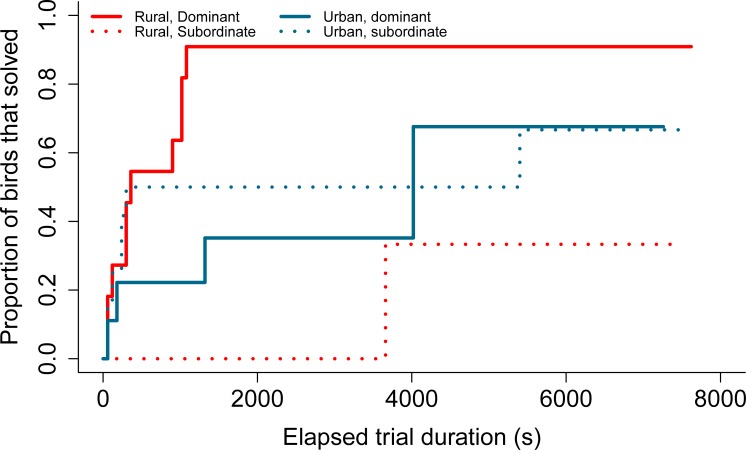
Relationship between dominance score and proportion of individuals that solved the lever-pulling task over two trials.

The lever-pulling task was solved by 54% (38/70) of individuals, 85% (33/38) of which solved in their first trial. The average latency to solve among solvers was 17.47 ± 5.10 SE minutes. When examining the determinants of performance in this task, our model selection procedure returned two top models (S5 Table). Dominance score and the interaction between dominance and habitat type were found to be important predictors of lever-pulling performance (Table 1A). These results suggest that dominance has a greater effect on lever-pulling performance in rural habitats, with more dominant individuals having a higher probability of solving the task compared to subordinates (Table 1A, Fig 3). Exploration score was the least important predictor of performance that was retained in the top models.

This plot was created with a model including only the interaction term between habitat and a categorical dominance variable. Each step in a line represents a bird solving the task, and a steeper line indicates greater problem-solving efficiency. Blue lines represent birds from urban sites and red lines represent those from rural sites. Dominant individuals are defined as those with an above average dominance score (solid lines), while subordinates have a below average dominance score (dashed lines). Sample sizes: rural, dominant = 11; rural, subordinate = 6; urban, dominant = 9; urban, subordinate = 12. Censored observations are not indicated.

**Table 1:** Model averaged estimates assessing the influence of predictors on (A) lever-pulling performance (n = 38 individuals, solutions = 22), (B) lever-pulling performance after replacing dominance with age and sex in the global model (n = 52 individuals, solutions = 33), and (C) persistence in the lever-pulling task (n = 39 individuals).

	*Parameter*	*Estimate*	*Standard Error*	*Confidence interval*	*Relative* *importance*
***A*** *Performance*	Habitat (stratified)	—	—	—	1.00
	Contacts	3.039	0.961	**(1.155, 4.923)**	1.00
	Dominance	5.251	2.065	**(1.204, 9.298)**	1.00
	Dominance* Habitat (Urban)	-4.825	2.328	**(-9.389, -0.262)**	1.00
	Exploration	0.803	0.896	(-0.953, 2.558)	0.60
***B***	Age(Adult)	0.969	0.409	**(0.167, 1.771)**	1.00
*Performance*	Contacts	3.862	0.661	**(2.566, 5.157)**	1.00
	Exploration	1.677	0.609	**(0.484, 2.871)**	1.00
	Sex(Female)	-0.101	0.258	(-0.605, 0.404)	0.29
*C*	Exploration	-0.491	0.553	(-1.592, 0.610)	0.59
*Persistence*	Urbanisation	0.064	0.201	(-0.337, 0.464)	0.16
	Dominance	-0.115	0.286	(-0.687, 0.456)	0.27

The reference levels for habitat, age, and sex are rural, juvenile, and male, respectively. Variables not retained in the set of top models are not shown (B–urbanisation, age*urbanisation, sex*urbanisation; C–latency to solve or censor, dominance*urbanisation). Confidence intervals that exclude zero are shown in bold text.

Three top models were returned after replacing dominance with age and sex in a new global model (S6 Table). We found an effect of age and exploratory tendency on the probability of solving the lever-pulling task (Table 1B). Adults had a higher probability of solving this task compared to juvenile birds, and more exploratory individuals were more likely to solve the task than less exploratory ones.

When examining the effects of individual characteristics on the total number of contacts made with the task (persistence), model selection returned five top models (S7 Table). The second-best fitting model was a null model, and none of the variables included were found to be important predictors after model averaging (Table 1C).

### Paper ripping

The paper-ripping task was solved by 41% (29/70) of individuals, 83% (24/29) of which solved in their first trial. The average latency to solve among solvers was 21.71 ± 6.27 SE minutes. When assessing the characteristics of innovative individuals, our model selection procedure returned four top models (S8 Table). Model averaging results showed no strong effects of the predictors of interest (Table 2A).

**Table 2:** Model averaged estimates assessing the influence of predictors on (A) paper-ripping performance (n = 33 individuals, solutions = 17), (B) persistence in the paper-ripping task (n = 36 individuals).

	*Parameter*	*Estimate*	*Standard Error*	*Confidence interval*	*Relative importance*
***A***	Contacts	23.438	4.584	**(14.454, 32.422)**	1.00
*Performance*	Dominance	-0.075	0.483	(-1.022, 0.872)	0.17
	Urbanisation	-1.756	1.450	(-4.598, 1.085)	0.79
	Exploration	0.167	0.646	(-1.100, 1.434)	0.20
***B*** *Persistence*	Latency to solve or censor	-1.871	0.410	**(-2.708, -1.035)**	1.00
	Urbanisation	-0.887	0.367	**(-1.635, -0.139)**	1.00
	Dominance	0.562	0.432	(-0.544, 0.939)	0.35

Variables not retained in the set of top models (A–dominance*urbanisation; B–exploration, dominance*urbanisation) are not shown. Confidence intervals that exclude zero are shown in bold text.

**Fig 4:**
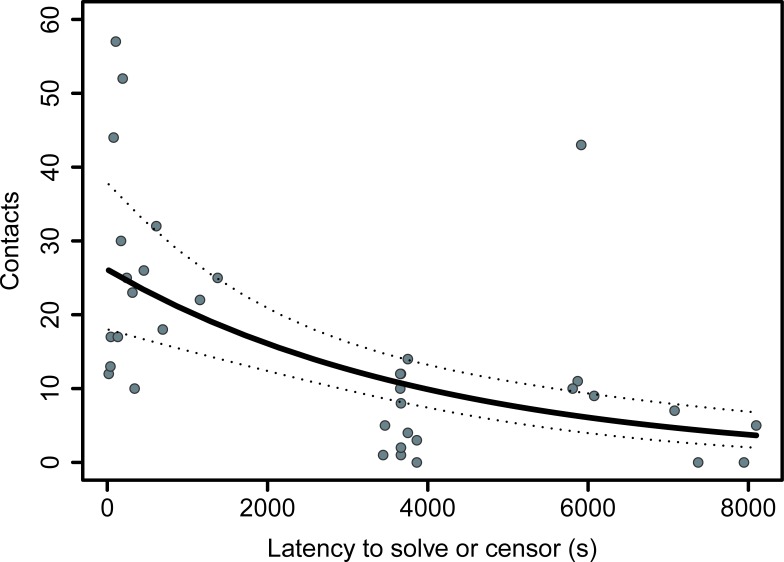
Relationship between number of contacts and latency to solve or censor in the paper-ripping task. The slope and 95% confidence interval reflect the model estimate for this variable when holding all other variables in the global model at their mean.

**Fig 5:**
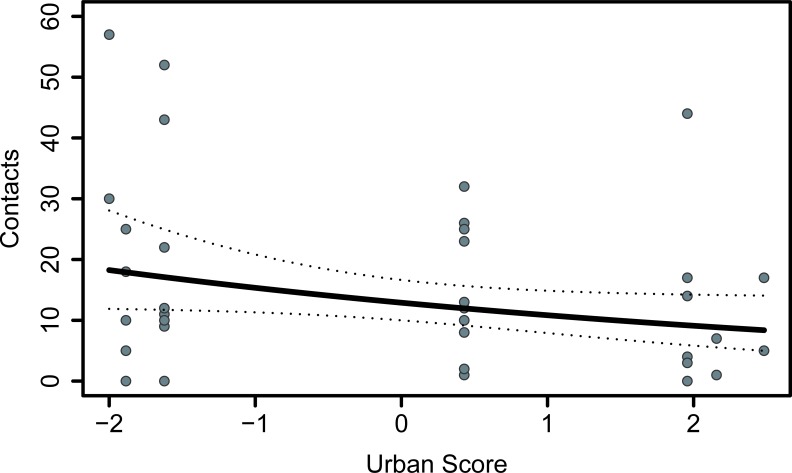
Relationship between number of contacts with the paper-ripping task and urbanisation of the habitat. A higher urban score corresponds to more urbanised sites. The slope and 95% confidence interval reflect the model estimate for this variable when holding all other variables in the global model at their mean.

Two top models (S9 Table) were returned by model selection using our global generalized linear mixed model examining the predictors of persistence in the paper ripping task. Both the latency to solution or censoring (Fig 4) and urbanisation score were negatively associated with the number of contacts with the task (Table 2B, Fig 5).

### Repeatability

We found no significant individual correlation in the latency to solve or censor (τ_b_ = 0.03, P = 0.71) or the total number of contacts (τ_b_ = 0.07, P = 0.45) between our two tasks. Additionally, our chi-square test of independence showed that the success in solving the lever-pulling task was not associated with success in the paper-ripping task (χ^2^ = 0.001, df = 1, P = 0.97).

## Discussion

Animal innovations have potential implications for the fitness of individuals, and the ecology and evolution of species, but the traits that characterize innovative individuals are not fully understood. We examined the predictors of innovativeness in black-capped chickadees using their performance on two food-motivated problem-solving tasks. We found that dominant individuals were more successful and efficient than subordinates in the lever-pulling task, especially if they were captured from rural forested patches. We also found evidence for a positive association between spatial exploration and lever-pulling performance. Furthermore, when analysing the predictors of persistence, we found that individuals originating from less urbanised habitats tended to have a greater total number of contacts with the paper-ripping task.

When assessing the effect of dominance rank on problem-solving performance, our results did not align with predictions from the necessity drives innovation hypothesis. In fact, our analysis suggested that higher ranking rural birds outperformed lower ranking individuals from rural sites in the lever-pulling task. This is contrary to previous findings that less competitive individuals tend to be more likely to innovate than the more competitive dominants, even when individuals are tested in an isolated context as in our study (e.g. [13]). Our result should be taken with caution since the interaction effect was only significant when excluding an influential observation (cf. Table A in S2 Table and Table A in S3 Table). However, as further evidence for this effect, dominants have previously been found to be more efficient in a lever-pulling task compared to subordinates in our population [67]. Assuming that lever pulling in captivity reflects variation in problem-solving performance on similar tasks in the wild (but see [68]), there could be two potential explanations for dominant individuals outperforming subordinates. The first possibility is that the costs of innovating vary between individuals of different competitive abilities. Less competitive individuals are at greater risk of losing newly discovered resources to conspecifics [69,70], thereby potentially decreasing the benefits of innovating. Alternatively, individuals of different competitive abilities may differ in the value that they place on available food items in isolated versus social contexts [13]. In a study where they determined competitive ability and innovativeness in captivity, O’Shea et al. [71] found that less competitive great tits were more likely to perform a string-pulling task in dyadic trials, but innovativeness in an isolated context was not associated with competitive ability. This suggests that less competitive individuals may forego a high-quality food resource available through novel means, but competition for established resources in a social context may drive them to innovate. If the costs of innovating are high for subordinate chickadees, or competition is required to elicit their problem-solving attempts, then we would predict that subordinate chickadees would interact with the lever-pulling task less than dominant individuals in the solitary context of our tests. However, dominance scores did not predict the level of persistence exhibited by individuals in the lever-pulling trials, so these explanations seem unlikely.

To investigate other explanations for this trend, we replaced dominance with age and sex in our model and found that adult chickadees outperformed younger individuals. This pattern matches findings in multiple species where adults tend to be more innovative than nonadults ([11,12,54], but see [68,72]). It may be that experience plays a role in the performance of individuals. Adult chickadees may have more information from their previous interactions with objects that can be applied to the new context of lever pulling. Another possibility is that adults employ a greater diversity of motor actions or contact a variety of task components when interacting with objects, which is known to predict innovation in some taxa [12,15,54,73]. Previous experience and/or greater motor diversity might allow adults to recognize functional components of a task or use the appropriate motor actions sooner than juveniles. The role of experience or motor tactics may be especially evident if juveniles interact with tasks more than adults, but still do not outperform them (e.g. in meerkats, [12]; and hyenas, [15]). There were few ways in which chickadees could manipulate each of our tasks, which would not allow for a meaningful analysis of motor diversity on their problem-solving performance.

The weaker effect of dominance on problem-solving performance in urban birds demonstrates the complexity with which urbanisation may impact problem-solving performance in animals, and may be explained by reduced competition between conspecifics resulting from the presence of ample resources year-round [6]. For example, a study on zenaida doves (*Zenaida aurita*) found that an urban population of these birds had begun to exhibit unaggressive scramble competition for food rather than interference competition as seen in other habitats [74]. In chickadees, both dominant and subordinate individuals from urban habitats may be less motivated to access novel resources, thereby explaining the steeper relationship between dominance and problem solving in rural birds. Further studies into the relationship between dominance and problem-solving performance would benefit from a deeper investigation into the behavioural and cognitive characteristics distinguishing individuals of varying dominance ranks, comparisons of problem solving in isolation versus in the presence of competitors, and larger sample sizes to enable robust conclusions.

When running our analyses with maximal sample size, we found, as predicted, that a greater tendency for spatial exploration is associated with a greater probability of solving the lever-pulling task. This result is in line with the findings of Overington et al. [17] and Perals et al. ([16], but see [19,75]) and provides support for the idea that exploratory tendency contributes to the innovative propensity of animals. Presumably individuals that show a greater tendency for spatial exploration are also more likely to approach and interact with novel objects (e.g. in common mynas [16]), although this is not always the case (e.g. in mountain chickadees [76]). The effect of exploration on problem-solving performance may result from our decision to analyse the full latency from the beginning of a trial to solution, so as to include all steps of the innovation process that animals would have to go through in the wild: detection, contact, and solution. The lack of an association between exploratory tendency and the number of contacts made with the lever-pulling task suggests that our exploratory chickadees may be faster to solve the task because they approach the task sooner (i.e. are less neophobic and/or more neophilic) rather than interacting with it more; this highlights the value of analysing predictors of contact rate to gain an understanding of the drivers of innovation at different stages of the process.

When assessing the predictors of the total number of contacts made with the tasks, we found that rural birds tended to contact the paper-ripping task more than urban individuals. However, this was not detected when excluding birds from sites with less transitive triads than expected and must therefore be interpreted with caution. This result is in contrast to a positive effect of urbanisation on the frequency of pecks directed at a problem by common mynas [23]. A possible explanation for this trend is that urban chickadees may not have a need to be persistent when foraging in the wild as they are likely to come across freely accessible resources such as bird feeders [25]. The presence of these resources may also make urban chickadees less likely to exhibit bark-pulling behaviour (foraging for food hidden under bark in natural environments, [28]), which resembles the motor actions needed to solve the paper-ripping task. In their study of Darwin’s finches, Tebbich et al. [77] attribute differences in problem-solving success between species to differences in natural foraging behaviour, where species using extractive foraging techniques in the wild are more successful in a problem-solving task. Foraging for hidden food may be a more natural behaviour for rural compared to urban birds, but this idea would need to be tested explicitly. Across species, individuals that persist in their interaction with a task, especially using appropriate motor actions, tend to be the most successful problem solvers (e.g. [5,54]), and those that persist in activities that may not be immediately beneficial are expected to be able to develop expertise in those behaviours [31]. Thus, further investigation of the characteristics associated with persistence is merited.

We found that an individual’s problem-solving success, efficiency, and persistence were not consistent across tasks. This inconsistency suggests that the protocols and characteristics of the problems used (e.g. visibility of the food reward, motor actions required to solve, food deprivation, and time spent in captivity) may have a significant impact on the relative performance of individuals. Our results are thus not generalizable to tasks that differ in multiple ways, but it may still be the case that they apply to performance on similar tasks. A study on house sparrows found that problem-solving performance was significantly repeatable across four tasks, but the tasks used all had visible rewards ([25], see also [19]). Griffin & Diquelou [21] also found that problem-solving success was consistent across three tasks with at least partially visible food rewards. Additionally, Van Horik & Madden [55] found that problem-solving success was consistent across two similar tasks, but not on a third one that differed in its structure. Tebbich et al. [78] propose that motor flexibility and goal-directed motivation may have a greater influence on problem-solving performance when the food reward is visible, and non-goal directed exploration may be more influential when food is hidden. So, problem solving may require different cognitive processes or behavioural tendencies depending on whether the food reward is detected by the animal or not. In the case of our tasks, we cannot determine which processes are at work as we did not have a meaningful measure of motor diversity and spatial exploration scores did not predict persistence in either task. Such potential influences of task characteristics may in part explain the mixed results of previous studies on individual and ecological predictors of problem solving in animals. Thus, an important step in determining the characteristics associated with innovativeness is to conduct an experimental assessment of the effect of task characteristics (i.e. using several replicates of tasks differing in each characteristic such as reward visible or not, type of motor action required, etc.) on individual performance and its repeatability.

In conclusion, we found a trend in rural birds for dominants to outperform subordinates in a lever-pulling task, which may be explained by adults outperforming younger birds. Moreover, we found that rural birds were slightly more persistent in the paper-ripping task compared to birds from urbanised areas. Individual performance and persistence were not repeatable across tasks, and the traits defining the most innovative and persistent individuals in each task were not consistent, pointing to the need for experimental assessments on the effect of task characteristics on the repeatability of problem-solving performance and persistence. Overall, our findings suggest that different individual and ecological characteristics may facilitate innovative behaviour in different ecological contexts.

## Supporting information

S1 Table **Model averaged estimates assessing the influence of predictors on (A) lever-pulling persistence (n = 39 individuals), (B) paper-ripping performance (n = 33 individuals, solutions = 17), and (C) paper-ripping persistence (n = 36 individuals).** The reference level for habitat is rural. Variables not retained in the set of top models are not shown (A–latency to solve or censor, dominance*habitat; B–dominance*habitat; C–exploration, dominance*habitat). Confidence intervals that exclude zero are shown in bold text.
(PDF)

S2 Table **Model averaged estimates assessing the influence of predictors on (A) lever-pulling performance (n = 39, solutions = 23) and (B) paper-ripping performance (n = 36, solutions = 20) prior to removing influential observations.** Variables not retained in the set of top models (B–dominance*urbanisation) are not shown. The reference level for habitat is rural. Confidence intervals that exclude zero are shown in bold text.
(PDF)

S3 Table **Model averaged estimates assessing the influence of predictors on (A) lever-pulling performance (n = 34, solutions = 20) and (B) paper-ripping performance (n = 32, solutions = 17) after removing individuals from sites that did not meet requirements for transitivity.** Variables not retained in the set of top models (B–dominance*urbanisation) are not shown. Confidence intervals that exclude zero are shown in bold text.
(PDF)

S4 Table **Model averaged estimates assessing the influence of predictors on (A) lever-pulling persistence (n = 34 individuals) and (B) paper-ripping persistence (n = 32 individuals) after removing individuals from sites that did not meet requirements for transitivity.** The interaction term was removed from the lever-pulling model due to convergence issues. Variables not retained in the set of top models (A–latency to contact or censor, urbanisation; B–exploration, dominance*urbanisation) are not shown.
(PDF)

S5 Table Top candidate models assessing the predictors of lever-pulling performance. (PDF)

S6 Table Top candidate models assessing the predictors of lever-pulling performance after replacing dominance with age and sex. (PDF)

S7 Table Top candidate models assessing the predictors of lever-pulling persistence. (PDF)

S8 Table Top candidate models assessing the predictors of paper-ripping performance. (PDF)

S9 Table Top candidate models assessing the predictors of paper-ripping persistence. (PDF)

S10 Table Field sites sorted by decreasing urbanization score. (PDF)

S11 Table First component correlation loadings from a principal components analysis used to generate an exploration score for each bird. (PDF)
