# Supplementary material for: Characterizing innovators: Ecological and individual predictors of problem-solving performance
Source: PLoS One. 2019 Jun 12;14(6):e0217464. doi: 10.1371/journal.pone.0217464 (PMC6561637; doi:10.1371/journal.pone.0217464)
Supplement: S1 Table — Model averaged estimates assessing the influence of predictors on (A) lever-pulling persistence (n = 39 individuals), (B) paper-ripping performance (n = 33 individuals, solutions = 17), and (C) paper-ripping persistence (n = 36 individuals). The reference level for habitat is rural. Variables not retained in the set of top models are not shown (A–latency to solve or censor, dominance*habitat; B–dominance*habitat; C–exploration, dominance*habitat). Confidence intervals that exclude zero are shown in bold text. (PDF) [file pone.0217464.s001.pdf]

	Parameter	Estimate	Standard Error	Confidence interval	Relative importance
A	(Intercept)	3.232	0.150	**(2.928, 3.536)**	--
	Exploration	-0.476	0.548	(-1.568, 0.616)	0.57
	Dominance	-0.119	0.289	(-0.698, 0.460)	0.28
	Habitat (Urban)	0.035	0.133	(-0.232, 0.302)	0.13
B	Contacts	22.795	4.325	**(14.319, 31.271)**	1.00
	Habitat (Urban)	-0.707	0.831	(-2.337, 0.922)	0.60
	Exploration	0.273	0.780	(-1.255, 1.802)	0.29
	Dominance	-0.071	0.455	(-0.964, 0.821)	0.13
C	(Intercept)	2.959	0.200	**(2.551, 3.367)**	--
	Latency to solve or censor	-1.950	0.417	**(-2.801, -1.099)**	1.00
	Habitat(Urban)	-0.643	0.271	**(-1.196, -0.091)**	1.00
	Dominance	0.144	0.324	(-0.505, 0.793)	0.30
