# Supplementary material for: Characterizing innovators: Ecological and individual predictors of problem-solving performance
Source: PLoS One. 2019 Jun 12;14(6):e0217464. doi: 10.1371/journal.pone.0217464 (PMC6561637; doi:10.1371/journal.pone.0217464)
Supplement: S2 Table — Model averaged estimates assessing the influence of predictors on (A) lever-pulling performance (n = 39, solutions = 23) and (B) paper-ripping performance (n = 36, solutions = 20) prior to removing influential observations. Variables not retained in the set of top models (B–dominance*urbanisation) are not shown. The reference level for habitat is rural. Confidence intervals that exclude zero are shown in bold text. (PDF) [file pone.0217464.s002.pdf]

	Parameter	Estimate	Standard Error	Confidence interval	Relative importance
A	Habitat (stratified)	--	--	--	1.00
	Contacts	3.286	0.969	**(1.387, 5.185)**	1.00
	Dominance	3.281	1.748	(-0.145, 6.706)	1.00
	Dominance*Habitat(Urban)	-2.531	2.247	(-6.934, 1.872)	0.74
	Exploration	1.094	0.933	(-0.736, 2.923)	0.74
B	Contacts	10.394	2.388	**(5.714, 15.073)**	1.00
	Dominance	0.176	0.545	(-0.892, 1.243)	0.28
	Urbanisation	-0.056	0.330	(-0.703, 0.590)	0.13
	Exploration	0.689	0.891	(-1.056, 2.435)	0.55
