# Supplementary material for: Characterizing innovators: Ecological and individual predictors of problem-solving performance
Source: PLoS One. 2019 Jun 12;14(6):e0217464. doi: 10.1371/journal.pone.0217464 (PMC6561637; doi:10.1371/journal.pone.0217464)
Supplement: S3 Table — Model averaged estimates assessing the influence of predictors on (A) lever-pulling performance (n = 34, solutions = 20) and (B) paper-ripping performance (n = 32, solutions = 17) after removing individuals from sites that did not meet requirements for transitivity. Variables not retained in the set of top models (B–dominance*urbanisation) are not shown. Confidence intervals that exclude zero are shown in bold text. (PDF) [file pone.0217464.s003.pdf]

	Parameter	Estimate	Standard Error	Confidence interval	Relative importance
A	Habitat (stratified)	--	--	--	1.00
	Contacts	2.811	1.184	**(0.492, 5.131)**	1.00
	Dominance	5.190	2.022	**(1.227, 9.153)**	1.00
	Dominance*Habitat(Urban)	-5.335	2.328	**(-9.898, -0.772)**	1.00
	Exploration	0.920	1.050	(-1.137, 2.977)	0.59
B	Contacts	17.306	3.401	**(10.640, 23.972)**	1.00
	Dominance	-0.079	0.485	(-1.030, 0.872)	0.17
	Urbanisation	-1.696	1.340	(-4.322, 0.931)	0.81
	Exploration	0.159	0.648	(-1.111, 1.430)	0.20
