# Supplementary material for: Characterizing innovators: Ecological and individual predictors of problem-solving performance
Source: PLoS One. 2019 Jun 12;14(6):e0217464. doi: 10.1371/journal.pone.0217464 (PMC6561637; doi:10.1371/journal.pone.0217464)
Supplement: S4 Table — Model averaged estimates assessing the influence of predictors on (A) lever-pulling persistence (n = 34 individuals) and (B) paper-ripping persistence (n = 32 individuals) after removing individuals from sites that did not meet requirements for transitivity. The interaction term was removed from the lever-pulling model due to convergence issues. Variables not retained in the set of top models (A–latency to contact or censor, urbanisation; B–exploration, dominance*urbanisation) are not shown. (PDF) [file pone.0217464.s004.pdf]

	Parameter	Estimate	Standard Error	Confidence interval	Relative importance
A	(Intercept)	3.235	0.147	**(2.935, 3.536)**	--
	Exploration	-0.279	0.515	(-1.305, 0.747)	0.33
	Dominance	-0.114	0.304	(-0.723, 0.494)	0.23
B	(Intercept)	2.537	0.152	**(2.225, 2.849)**	--
	Latency to solve or censor	-1.730	0.481	**(-2.714, -0.745)**	1.00
	Urbanisation	-0.378	0.465	(-1.307, 0.551)	0.52
	Dominance	0.247	0.441	(-0.635, 1.130)	0.37
