# Supplementary material for: Characterizing innovators: Ecological and individual predictors of problem-solving performance
Source: PLoS One. 2019 Jun 12;14(6):e0217464. doi: 10.1371/journal.pone.0217464 (PMC6561637; doi:10.1371/journal.pone.0217464)
Supplement: S5 Table — (PDF) [file pone.0217464.s005.pdf]

Model	Predictors	df	logLik	AICc	ΔAICc	ω_i
1	Contacts + dominance + exploration + habitat(stratified) + dominance*habitat(stratified)	4	-40.251	88.5	0.00	0.6
2	Contacts + dominance + habitat(stratified) + dominance*habitat(stratified)	3	-41.661	89.3	0.81	0.4
