# Supplementary material for: Characterizing innovators: Ecological and individual predictors of problem-solving performance
Source: PLoS One. 2019 Jun 12;14(6):e0217464. doi: 10.1371/journal.pone.0217464 (PMC6561637; doi:10.1371/journal.pone.0217464)
Supplement: S6 Table — (PDF) [file pone.0217464.s006.pdf]

Model	Predictors	df	logLik	AICc	ΔAICc	ω_i
1	Age + contacts + exploration	3	-94.566	195.1	0.00	0.512
2	Age + sex + contacts + exploration	4	-94.135	196.3	1.14	0.289
3	Age + contacts + exploration + urbanisation	4	-94.510	197.0	1.90	0.199
