# Supplementary material for: Characterizing innovators: Ecological and individual predictors of problem-solving performance
Source: PLoS One. 2019 Jun 12;14(6):e0217464. doi: 10.1371/journal.pone.0217464 (PMC6561637; doi:10.1371/journal.pone.0217464)
Supplement: S7 Table — (PDF) [file pone.0217464.s007.pdf]

Model	Predictors	df	logLik	AICc	ΔAICc	ω_i
1	Exploration	4	-164.663	338.5	0.00	0.308
2	NULL	3	-166.080	338.8	0.34	0.260
3	Exploration +urbanisation	5	-163.998	339.8	1.31	0.160
4	Dominance	4	-165.366	339.9	1.41	0.153
5	Dom + explor	5	-164.293	340.4	1.90	0.119
