# Supplementary material for: Characterizing innovators: Ecological and individual predictors of problem-solving performance
Source: PLoS One. 2019 Jun 12;14(6):e0217464. doi: 10.1371/journal.pone.0217464 (PMC6561637; doi:10.1371/journal.pone.0217464)
Supplement: S8 Table — (PDF) [file pone.0217464.s008.pdf]

Model	Predictors	df	logLik	AICc	ΔAICc	ω_i
1	Contacts + urbanisation	2	-18.138	40.3	0.00	0.426
2	Contacts	1	-19.853	41.7	1.43	0.209
3	Contacts + exploration + urbanisation	3	-17.910	41.8	1.55	0.196
4	Contacts + dominance + urbanisation	3	-18.059	42.1	1.85	0.169
