# Supplementary material for: Characterizing innovators: Ecological and individual predictors of problem-solving performance
Source: PLoS One. 2019 Jun 12;14(6):e0217464. doi: 10.1371/journal.pone.0217464 (PMC6561637; doi:10.1371/journal.pone.0217464)
Supplement: S9 Table — (PDF) [file pone.0217464.s009.pdf]

Model	Predictors	df	logLik	AICc	ΔAICc	ω_i
1	Contacts + urbanisation	5	-128.623	269.2	0.00	0.648
2	Contacts + dominance + urbanisation	6	-127.787	270.5	1.22	0.352
