# Supplementary material for: Characterizing innovators: Ecological and individual predictors of problem-solving performance
Source: PLoS One. 2019 Jun 12;14(6):e0217464. doi: 10.1371/journal.pone.0217464 (PMC6561637; doi:10.1371/journal.pone.0217464)
Supplement: S10 Table — (PDF) [file pone.0217464.s010.pdf]

Habitat	Site Code	Urban Score	Latitude, Longitude	N
Urban	AP	2.48	45°25'40"N, 75°37'49"W	6
	CP	2.16	45°24'32"N, 75°39'08"W	6
	FRP	1.96	45°21'26"N, 75°47'18"W	10
	ML	0.43	45°22'11"N, 75°47'46"W	12
				Total = 34
Rural	BB	-1.62	45°11'34"N, 76°18'3"W	12
	SR	-1.89	45°20'56"N, 75°23'11"W	12
	LNP	-2.00	45°26'55"N, 75°11'11"W	12
				Total = 36
