# Supplementary material for: Characterizing innovators: Ecological and individual predictors of problem-solving performance
Source: PLoS One. 2019 Jun 12;14(6):e0217464. doi: 10.1371/journal.pone.0217464 (PMC6561637; doi:10.1371/journal.pone.0217464)
Supplement: S11 Table — (PDF) [file pone.0217464.s011.pdf]

Variables	PC1 Loadings
Visits to tree 1	0.92
Visits to tree 2	0.90
Visits to tree 3	0.95
Visits to tree 4	0.92
Other features	0.54
Duration of flights	0.91
Duration of hops	0.89
% variation explained	75.81%
Eigenvalue	2.30
